# A promising device to save maternal lives associated with obstetric hemorrhage: the non-pneumatic anti-shock garment (NASG)

**DOI:** 10.1186/s12978-015-0019-6

**Published:** 2015-03-31

**Authors:** Suellen Miller, José M Belizán

**Affiliations:** Director Safe Motherhood Programs, University of California, San Francisco, School of Medicine, Department of Obstetrics, Gynecology, and Reproductive Sciences, California, USA; Institute for Clinical Effectiveness and Health Policy, Buenos Aires, Buenos Aires, Argentina

**Keywords:** Hemorrhage, Hypovolemic shock, Obstetric first-aid, Shock garment

## Abstract

ᅟ

Preventable maternal mortality associated with obstetric hemorrhage is a human tragedy that mainly occurs in low and middle-income countries (LMIC). Massive blood loss leads to hypovolemic shock, which decreases blood flow and therefore oxygen to tissues in the heart, lungs, and brain. Women can rapidly develop disseminated intravascular coagulopathy, tissue anoxia, single or multiple organ failure, and death. Management and treatment of hypovolemic shock due to massive obstetric hemorrhage requires care at the tertiary or referral level, treatment varies dependent on hemorrhage etiology, but generally reversing shock requires not only uterotonics and IV fluids (sometimes available at primary health centers), but also blood transfusions and hemostatic surgeries, only available at Comprehensive Emergency Obstetric Care (CEmOC) facilities.

The Non-Pneumatic Anti-Shock Garment (NASG) is a first aid device that can buy time for women experiencing hypovolemic shock secondary to obstetric hemorrhage from any etiology. As such it buys time to overcome two of the three delays that contribute to maternal mortality in lower resourced settings [1]. The second delay is a delay in arranging transport and during transport from either home or lower level health care facilities to referral facilities, and the third delay is the delayed receipt of quality, comprehensive emergency obstetrical care at the tertiary facility. The NASG can mitigate both these delays [2].

In the article published within *Reproductive Health*, the authors, all international, maternal health researchers, are publishing the results of a systematic review of six studies on the NASG conducted in LMICs [3]. Four studies are quasi-experimental, pre-intervention/intervention design in Egypt [4,5], Nigeria [6], and Zimbabwe [7] and one was a non-randomized concurrent comparative study in India [8]. These studies were conducted at the tertiary level, and compared outcomes of women with hypovolemic shock treated with standardized hemorrhage/shock protocols with similar women treated with the same protocols, plus the NASG. One study, set in Zambia and Zimbabwe [9] was a cluster-randomized control trial (c-RCT) which compared outcomes for women who either received the NASG prior to transport from primary health care center to tertiary level with outcomes for women who received the NASG after arrival at the tertiary level.

Systematic review and meta-analytic techniques were used to pool the results from the quasi-experimental studies. Use of these techniques is important when dealing with small samples and rare events. As the authors note, while maternal hemorrhage is a leading cause of maternal death, accounting for over 27% of all maternal deaths, and is associated with a huge burden of global disease; at the facility level life-threatening maternal hemorrhage and shock are rare events. Therefore, most studies use proxies for mortality or severe morbidity.

This systematic review notes appropriately that these NASG studies are unusual in using mortality and severe morbidity as their direct outcomes. The findings of the review of the pooled 5 quasi-experimental studies have an overall sample size of 2,330 women with severe hemorrhage, and a statistically significant decrease in pooled risk ratio (RR) of maternal mortality of 48% (RR 0.52 (95% CI: 0.36-0.77)). The single c-RCT with a smaller sample size and rare event rate, showed a non-significant statistical reduction in mortality, RR 0.43 (95% CI; 0.14-1.33. Finding a greater than 50% reduction in mortality for any treatment is a major impact and this finding deserves serious consideration for implementing the NASG innovation in maternal health care.

As the authors note the NASG appears to be safe, low cost, and easy to use, easy to train, with a potential for mitigating blood loss; the studies together show a trend towards beneficial effects from this device.

The c-RCT showing a non significant 57% mortality reduction examined a very short time frame [9]. Rather than comparing women who received NASG to women who did not receive the NASG, the study examined the effect of earlier application of NASG and its use during transport. This is an important distinction given that many women, who die from hemorrhage, die en route to tertiary centers or arrive at the tertiary centers too late for resuscitation. The time difference for earlier NASG application in these peri-urban clinics was less than 2 hours. Women who had the NASG applied < 2 hours before the “control group” who got NASGs at the referral center had a 57% decreased risk of dying from hypovolemic shock. This study had planned to enroll 2400 women for statistical power, but was closed for financial reasons when only 880 women were enrolled.

The NASG has been included in the WHO Guidelines for prevention and management of PPH [10] and in the International Federation of Obstetrics and Gynecology PPH Guidelines [11].

The NASG is currently being used in at least 20 countries globally, including the UK and USA. Examples include scaling up projects, in Ethiopia and implementation research studies of scale-up in Timor Leste and Tanzania. NASGs are incorporated into hypovolemic shock management protocols for transport or for overcoming delays in management (lack of blood, shortage of qualified personnel, such as surgeon or anesthesiologists). In Tamil Nadu, India, ambulance drivers are learning to apply NASGs.

The authors of the review note some limitations to the NASG studies, to their review, and to the potential for scale up of NASGs: issues with cleaning, reuse, exchange and return of NASGs between levels of the system. Advancements are underway in the implementation and the use of NASGs. The University of California, San Francisco (UCSF) has completed clinical research and product testing to develop a reduced bleach concentration cleaning protocol (0.01% solution) and to establish a performance standard for the NASG. After multiple uses and cleanings, field-testing is conducted according to a simplified 15-minute protocol using a blood pressure cuff and a healthy woman to verify that the NASG still meets the necessary compression performance standards. High quality, lower cost manufacturing has led to a decrease in cost and increase in durability.

These improvements in durability and specifications for cleaning and re-use, combined with the increased affordability of the NASG, are intended to support roll-out of this innovative product within the PPH continuum of care in low- and middle-income countries.

We agree with the authors of the review that the evidence demonstrates that the NASG is a promising intervention to stabilize women with severe maternal hemorrhage until they can receive definitive care. We consider that its use is warranted and we recommended its deployment in a context of implementation research studies or in program implementation with rigorous monitoring in order to continue to document NASG’s effects on maternal outcomes and on health systems strengthening. We further agree with the authors of the review that countries need to invest in definitive therapies, train more health care workers/skilled attendants, and provide access for all women to blood transfusions, surgery and to high quality, respectful, evidence-based care. However, including the NASG as a first-aid device is not an either/or, zero-sum strategy; the NASG is a first-aid device to reverse shock, and as such is a supplement to, not a replacement for, definitive treatment. Until every woman has immediate access to life saving shock and hemorrhage management this simple, low-cost device can be used to buy time and save lives.
